# Metal–Ceramic Compatibility in Dental Restorations According to the Metallic Component Manufacturing Procedure

**DOI:** 10.3390/ma16165556

**Published:** 2023-08-10

**Authors:** Nazem Dawod, Marian Miculescu, Iulian Vasile Antoniac, Florin Miculescu, Doriana Agop-Forna

**Affiliations:** 1 Faculty of Material Science and Engineering, University Politehnica of Bucharest, 313 Splaiul Independentei, District 6, 060042 Bucharest, Romania; dawod.nazem@gmail.com (N.D.); iulian.antoniac@upb.ro (I.V.A.); florin.miculescu@upb.ro (F.M.); 2 SC Sesene Dent SRL, 37 Vulcan Judetul Street, District 3, 030055 Bucharest, Romania; 3 Academy of Romanian Scientists, 54 Splaiul Independentei, 050094 Bucharest, Romania; 4 “Gr.T. Popa” University of Medicine and Pharmacy, 16 Universității Street, 700115 Iași, Romania; doriana.agop-forna@umfiasi.ro

**Keywords:** metal–ceramic systems, dental restorations, compatibility, thermal expansion, three-point bending

## Abstract

In terms of production technology, metal–ceramic systems for dental restorations comply with a concrete algorithm, the efficiency of which is always dependent on the applications for which they are intended. The first stage involves obtaining metal support, followed by firing the ceramic on the surface of the metal to meet the list of functional and aesthetic requirements of a future restoration. The compatibility of the two materials—the metal component and the ceramic component—must be ensured in several respects: chemical compatibility, thermo–chemical compatibility, and mechanical compatibility. Thus, there is a need to simulate the thermal behavior of the metal–ceramic couple in its processing to achieve appropriate dental prostheses. In this study, three types of Co–Cr metal frames were manufactured using three different production technologies: conventional casting, milling (CAM), and selective laser melting (SLM). Composition analyses, scanning electron microscopy (SEM), and microstructural analyses of the metal–ceramic interface for each type of production technology, as well as the determination of the hardness and the thermal expansion coefficients of experimental materials and three-point bending tests, were carried out in this study. Considering all these aspects, we demonstrated the influence of the technology of producing the metallic part of the metal–ceramic bonding process in dental prostheses.

## 1. Introduction

Metal–ceramic systems are used for dental restorations with the aim of combining the properties of metal alloys, such as those with high tensile strength, compressive strength, wear resistance, and corrosion resistance, with aesthetic appearance that is naturally provided by ceramic materials [1]. The systems obtained from these two types of materials have been successfully used in recent years, thanks to their ability to meet the functional requirements of dental restorations and, at the same time, offer excellent aesthetic results [2,3]. Their good corrosion behavior, especially under the influence of liquids in the oral cavity as well as their biocompatibility with oral tissues, have made them one of the most demanded options on the market [4,5].

Regarding their vast applicability in the dental field, one of the main applications of metal–ceramic systems in dentistry is in the manufacture of dental crowns and bridges. The combination of metal and ceramic allows precise control over the hue, translucency, and texture of the restoration surface, allowing the restoration result to blend seamlessly with the patient’s natural dentition.

Another application supported by metal–ceramic systems is the construction of prostheses on implants. The metal substructure provides the necessary strength to withstand occlusal forces, while the ceramic layer provides a natural look, imitating the optical properties of neighboring teeth. In addition to these benefits, metal–ceramic biocompatibility minimizes the risk of allergic reactions and adverse tissue responses, ensuring long-term success and patient satisfaction [6,7,8].

The connection between metal and ceramic ensures the simultaneous operation of the two materials to provide the strength and durability necessary for a dental restoration, so that it can be subjected to cutting and mastication forces in the oral cavity [9,10,11]. A strong bond prevents the chipping or cracking of ceramics, which can lead to failure of a restoration over time.

An important aspect to consider for the good functioning of metal–ceramic systems, as well as for the long-term success of a restoration, is the strength of the bond between the two materials. The retention or adhesion of metal to various substrates, such as enamel, dentin, ceramics, and old resins, is crucial in ensuring a successful restoration. In today’s aesthetic-focused dental practices, clinicians demand multifunctional primers or adhesives that can provide strong and durable adhesion [12,13,14].

The metal component in porcelain-fused-to-metal (PFM) restoration systems plays a crucial role in providing overall support and strength to a restoration. The metal substructure is usually made up of a combination of precious or non-precious metals, such as gold, silver, palladium, or cobalt–chromium alloy [5]. In addition, the metal surface must be properly prepared so that porcelain may properly stick to it. The ceramic component can completely or partially cover the metal component. This combination is common in clinical applications, such as removable prostheses, which use friction with parallel hard surfaces and internal clearances of adjacent teeth, respectively, and dental implants. The use of alloys of base metals makes it possible to provide high-quality treatment for many patients with limited financial means. Currently, the most widely used base-metal alloys for metal–ceramic restorations are nickel–chromium (Ni–Cr) materials. However, potential health problems associated with beryllium and nickel have led to the development of cobalt–chromium (Co–Cr) alloys [15].

In terms of production technology, metal–ceramic systems comply with a concrete algorithm, the efficiency of which is always dependent on the applications for which they are intended. The first stage involves obtaining metal support, followed by firing the ceramic on the surface of the metal to meet the list of functional and aesthetic requirements of a future restoration [16].

The compatibility of the two materials—the metal component and the ceramic component—must be ensured in several respects: chemical compatibility, thermo–chemical compatibility, and mechanical compatibility. Without such compatibility, there is a risk of cracks in the ceramic, deformation of the metal support, or undesirable aesthetics of the restoration caused by the ceramic part [16]. Chemical compatibility depends on the chemical composition of the opaque layer and the type of metal oxides formed at the interface. Thermo–mechanical compatibility depends on the thickness of the layers, differences in thermal expansion coefficients, the firing temperature of the ceramic, resistance of the metal to high temperatures, and exchanges of internal stresses. 

Regarding mechanical compatibility, the development of a metal–ceramic system involves four important mechanisms during the firing and sintering of the ceramic: the formation of chemical bonds, the occurrence of mechanical interlocks, van der Waals forces (which contribute to a small extent by forming interatomic forces), and compression forces. The process of forming chemical bonds is the first and most important of these mechanisms because such bonds establish a thermodynamic equilibrium between the two materials by forming an intermediate oxide layer [17].

In terms of mechanical interlocking, the outer surface of the metal exerts compressive forces that provide the ceramic with stable support. Mechanical interlocking depends on the surface roughness of the metal material, and can lead to the formation of gaps at the interface (microrets) that aggressively affect the bond between the metal and the ceramic [18,19].

Thermo–mechanical stresses and transient thermal stress are caused by differences, during the combustion cycle, in the temperature range for thermal contraction and thermal expansion of the metal alloy and the ceramic [20]. The existence of transient and residual thermal stresses depends on the thermal compatibility of the porcelain and the alloy. Unless these transient thermal stresses are accompanied by alloy creep or porcelain cracking, they are not harmful. Additional residual stresses may result from varying cooling rates or irregular porcelain/metal thickness ratios. A successful connection between the two materials involved in the restoration process implies a high resistance to the combination of residual thermal stresses and mechanical stresses that are applied [21].

Differences between metals and ceramics in the coefficients of thermal expansion lead to the occurrence of residual stresses in the ceramic mass, even before the application of a compressive force. The alloy must have a coefficient of thermal expansion higher than that of the ceramic, i.e., a positive value of the difference in coefficients, to produce compression in the ceramic mass when cooling. The ideal variation is between 0.5 and 1.0 × 10^−6^ °C^−1^, which permanently exerts compressive forces in a ceramic, thereby increasing the life of a dental restoration [22,23].

To protect a ceramic from cracking or fracturing due to thermally induced tensile stresses, the ceramics must be in slight compression before final adjustments. To induce such compression during the combustion process, the coefficient of thermal expansion of the metal should, in principle, be slightly higher than that of the ceramic during the cooling process at room temperature. Such a small difference causes a certain amount of pressure in the ceramic and, thus, strengthens the restoration [21].

In the case of metal–ceramic restorations, the thermal expansion coefficients of the ceramic and the metal are of particular interest in the temperature range of 600–625 °C or 500–525 °C. Metal–ceramic systems that have a difference in thermal expansion coefficients (metal minus ceramic) of 0.5 × 10^−6^/°C or less are acceptable [20]. According to some experiments, a slightly larger difference, up to 0.75 × 10^−6^/°C, can be used without the risk of cracks or fractures in the ceramic [22].

In most cases, manufacturers of dental restoration materials provide general or greatly simplified results or data on the material properties of such materials. In this study, we provide a series of application results in an explicit form—for example, whether and how the results depend on the structure or properties of the materials [24]. Understanding and predicting the final structure of any alloy and/or its subsequent behavior in operation requires, among other things, a determination of its many properties. This information is required, for example, as input into the numerical modeling of a restoration process. In most cases, such modeling is followed by a simulation of the studied process, tracking the evolution of some parameters with the help of the model under conditions as close as possible to the real conditions. This paper considers the applicability of a combination of methods of material investigation in the study of metal–ceramic compatibility. 

The idea behind this study arose from the need to simulate the thermal behavior of the metal–ceramic couple, during processing, to achieve appropriate dental prostheses that were made necessary because of multiple failures (cracks, delamination, etc.) that appeared after ceramic coatings. This approach involves microstructural analyses for each type of production technology for the metal–ceramic interface, together with a determination of the thermal expansion coefficients of the experimental materials and application of three-point bending tests. The dilatometry tests, on the one hand, simulate the behavior of experimental metal alloys during ceramic coating and, on the other hand, provide real technological solutions to avoid their failure (as reported by dentists).

Considering all these aspects, in this study, we demonstrate the influence of the technology of producing the metal part on dental prostheses and propose a modification of that technology, depending on the production technology of the metal component.

## 2. Materials and Methods

Three types of Co–Cr metal frames were manufactured using three different production technologies: conventional casting, computer-aided manufacturing (CAM) milling, and selective laser melting (SLM). The Co–Cr alloy was a commercial one (Heraenium^®^ P—Kulzer GmbH, Hanau, Germany) with its chemical composition specified by the producer, as presented in Table 1. 

From each sample according to the manufacturing technology type, we prepared samples for subsequent characterization: microstructural characterization, compositional analyses, thermal expansion, hardness testing, metal ceramic interface, and a three-point bending test. For the metal ceramic interface and the three-point bending test, each sample was ceramic-coated with HeraCeram (Kulzer GmbH, Hanau, Germany), according to the producer’s specifications, at a laboratory focused on dental techniques and dental equipment. The procedures for ceramic firing were identical, regardless of the technology used for production of the metal component. These procedures were applied after the standard preparation steps for the metal samples and their degassing at a temperature of 980 °C in vacuum for 7 min, marked by the formation of a film of yellow or slightly greenish oxides. The opaque film was applied directly to the oxides, in a thin layer, followed by burning at 850 °C for 10 min. Finally, dentin was applied in two layers and burned at 950 °C for 8 min. The glazing layer was not applied, in accordance with the standard requirements. 

**Figure 1:**
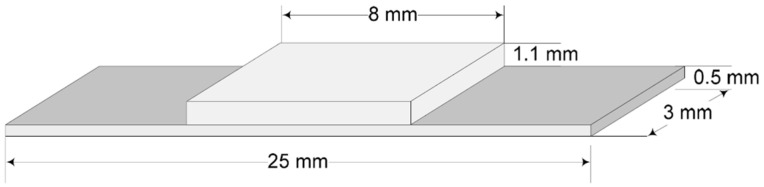
Three-point bending test on sample preparation sketch.

For the microstructural characterization and interface analysis, the samples were metallographically prepared in the following steps: a representative sample of the material was selected to properly characterize the microstructure and the features of interest; the test sample was sectioned carefully to avoid altering or destroying the structure of the material, using a Buehler Abrasimet^TM^ Delta cutter (Buehler Ltd., Lake Bluff, IL, USA) with an abrasive disc (102510P Buehler abrasive blade), under cooling, the deformation induced removal during subsequent sample preparation steps; the specimen was hot-mounted in PhenoCure^TM^ phenolic thermoset resin that provided good edge retention and moderate shrinkage, to facilitate handling during the grinding and polishing steps; the grinding was made using water-lubricated CarbiMet^TM^ silicon carbide grinding paper with grit sizes from P180 to P1000s; and the polishing steps were carried out, according to the Buehler SumMet method for cobalt alloys [22]. The samples were etched using a 2% Nital solution, then optical microscopy images were acquired using a Nikon Optical Microscope (Nikon Corporation, Minato, Tokyo, Japan) with NIS-Elements software 3.01. Sheets of Co–Cr alloys from each type were made, according to ISO 9693 indications, with sizes of (25 ± 1) mm × (3 ± 0.1) × mm (0.5 ± 0.05) mm. The schematic of the samples is presented in Figure 1 [25]. A set of 9 test samples, 3 from each type of manufacturing, were obtained.

Three-point bending tests were performed on the metal–ceramic samples, according to ISO9693 [23], using a mechanical-testing machine with a cell force of 10 KN (Instron 3400, Instron, Norwood, MA, USA), and a crosshead speed of 1.5 mm/min was applied. The distance between the supports was 20 mm and the diameter of the bending lever was 5 mm.

A universal hardness tester, Wilson UH4250 (Buehler, Germany), was used for the automatic HV5 hardness determination, according to ISO 6507–1 [26], using the Diamet^TM^ software on 3 samples of each type and 5 idents on each type.

For the dilatometric study, cylindrical samples of approximately 50 mm in height and 4 mm in diameter were made from each type of manufactured alloy and from the dentin and the enamel. The testing was carried out for the metallic samples in the range of room temperature (RT) to 1350 °C temperature, at both heating and cooling, and for the ceramic samples in the range of RT to 900 °C using a Unitherm 1611 (Anter Co., Pittsburgh, PA, USA) vertical dilatometer at a 2 °C/min heating/cooling rate. The coefficients of thermal expansion (CTEs) for both the metallic and ceramic parts were calculated and compared.

For the SEM and energy-dispersive spectroscopy (EDS) analyses, a Quanta S STEM (Thermo Fisher Scientific Inc., Waltham, MA, USA) microscope, equipped with an EDS detector, was used.

## 3. Results

### 3.1. Optical Microscopy

**Figure 2:**
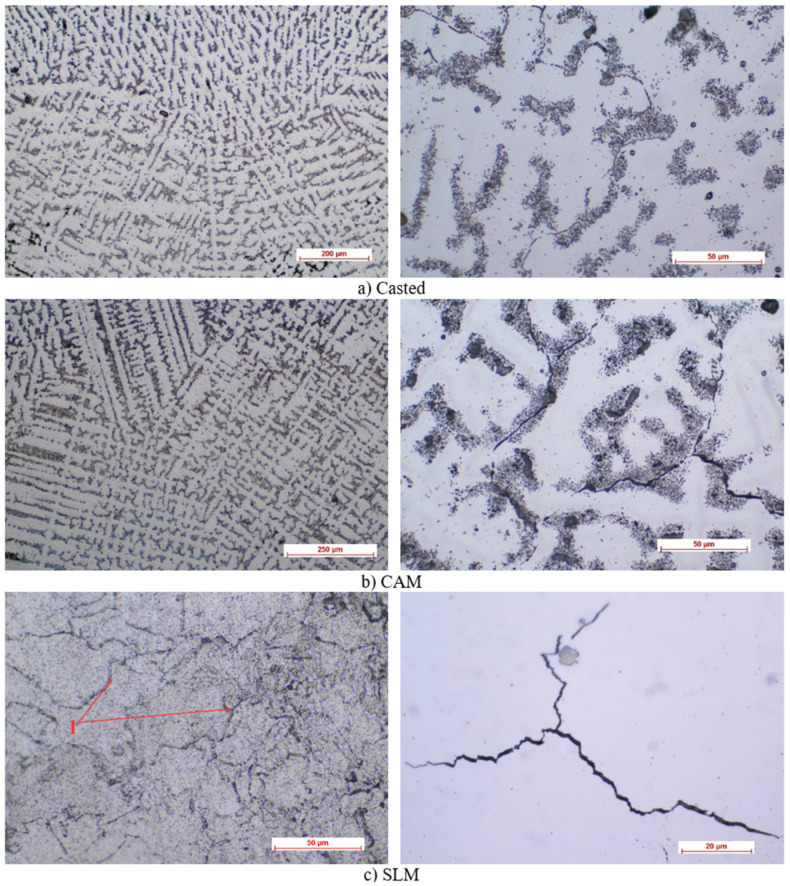
Optical microscopy images of the metal component (1—particle limits).

The optical microscopy results showed, for the specimens obtained by casting (as shown in Figure 2), a heterogeneous, dendritic microstructure, with a multitude of defects, following the production procedure. There was also a large volume of pores, which could be a precursor for internal stresses that caused the bending strength of the cast Co–Cr alloy to be low.

For specimens obtained by milling, an improvement in the appearance of the micrography was observed, in the form of a slightly more homogeneous structure than that of the cast samples. However, the same dendritic appearance was preserved, accompanied by random black dots that marked the presence of microgrids.

The chemical attack at the particle boundary (not grains, but sintered particles) was easiest to visualize in the case of sintered alloys (the particles limits of which were slightly over-attacked). The dimensions of the particles were completely uneven and ranged from 20 to 150 microns. Due to sintering, the particles occupied a smaller volume and conglomerates appeared, but there were also some areas that were not fully sintered, in terms of voids.

**Figure 3:**
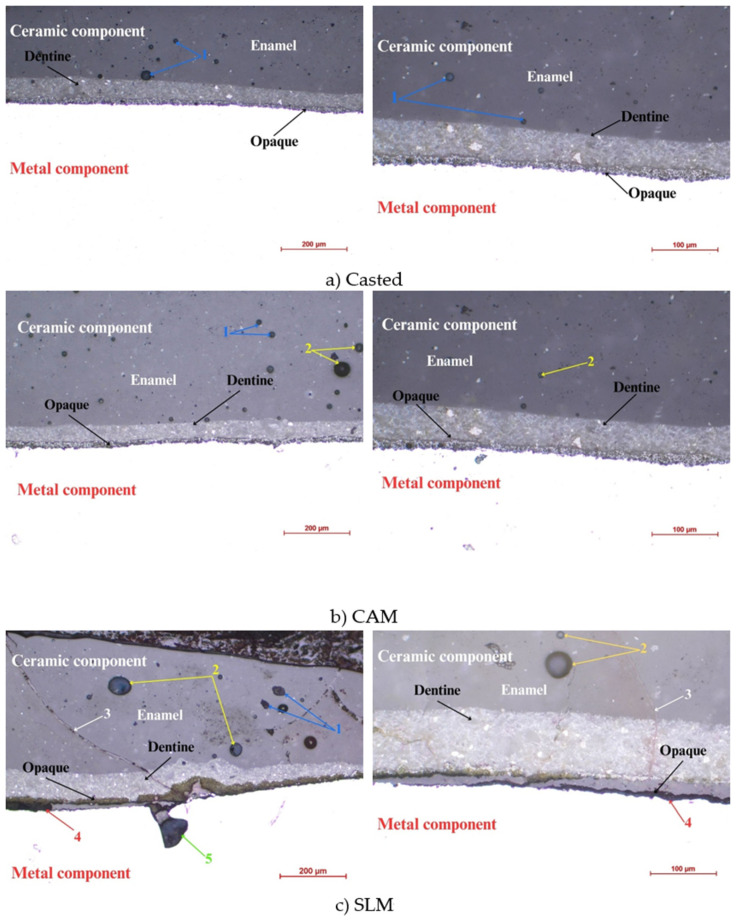
Optical microscopy images of metal–ceramic interfaces’ function on the production technology.

Figure 3 illustrates metal–ceramic interfaces according to the type of metal support. The metal components are marked at the bottom on the white background and the ceramic components are marked at the top. Between the two components are arrows that depict, in the order of the deposit on the metal component, the opaque layer, the dentin layer, and the enamel layer. The blue color and the number 1 represent the defects, the inclusions, and the voids in the ceramic material; the yellow color and the number 2 represent the pores in the ceramic component. For the sintered part, the deposited ceramic has a crack (number 3) shown by white in Figure 3, and poor adhesion between the two components (4—red), as well as a visible defect in the metal part (5).

A uniform sequence of layers, an almost perfect adhesion (Figure 3), and the fewest defects in both the ceramic and the metal mass (Figure 2), are characteristics of the casting parts. 

### 3.2. SEM/EDS

The scanning electron microscopy images complete the metallographic aspects presented above, both morphologically and compositionally.

**Figure 4:**
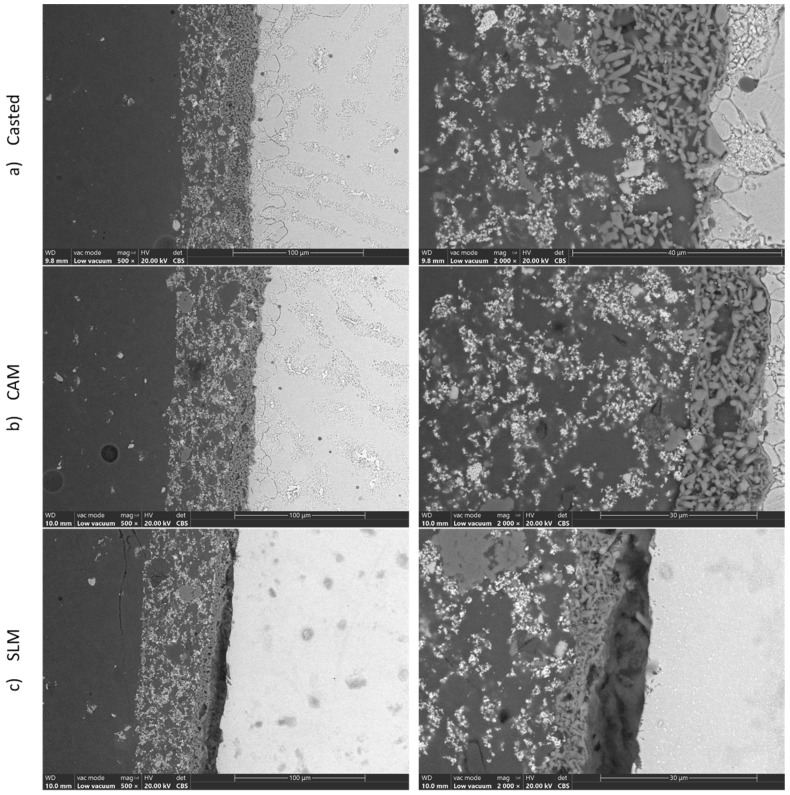
SEM images of the metal ceramic interfaces of (**a**) casted alloy sample, (**b**) milled alloy sample, and (**c**) sintered alloy sample.

Compositionally, the alloy was within the manufacturer’s standard. On the microzone that was analyzed to determine the chemical composition, a slight deviation from the composition of the manufacturer’s standard was observed, due to the strong micro-segregation of the material. Images from Figure 4a,b reveal the existence in the interdendritic space, in a proportion of less than 10%, of an intermetallic compound based on Co–Cr—probably Co3Cr, which is formed at a percentage of 23% Cr. The intermetallic compound was placed in phase ζ, which had a higher percentage of Cr (50.5–63%), compared to the majority dendritic structure of αCo. Due to the small size of both the interdentritic space and the compounds placed in it, the EDS method could not quantitatively identify the intermetallic compound and the ζ phase. The assessment was strictly a qualitative one, based on the image and the correlation with the equilibrium diagram. The backscattered electron image of the metal–ceramic interface, shown in Figure 4a,b, highlights the perfect adhesion of the ceramics to the metallic support; the continuity and lack of pores are highlighted, as well as the homogeneity of the deposited ceramics. Figure 4c shows an interruption in the contact of the deposited ceramic mass.

### 3.3. Thermal Expansion

**Figure 5:**
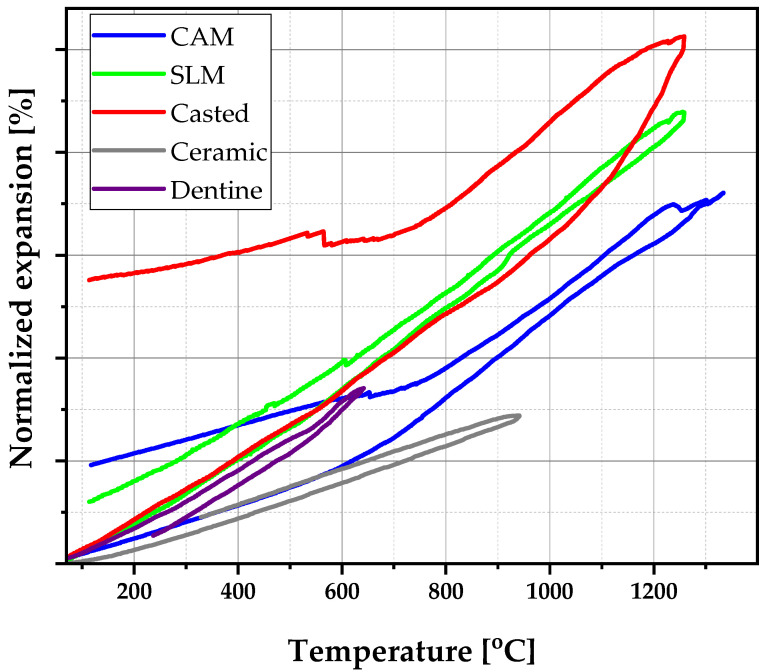
Comparative image of linear thermal expansion for the CoCr sample, obtained via different methods for the PFM ceramic and dentine.

The temperature range of interest in this study was 600 °C to 900 °C, where—correlated with literature values—the coefficient of thermal expansion of the alloy obtained by CAM was the most appropriate (see Figure 5 and Table 2). It was necessary that the metal and porcelain had identical coefficients of thermal expansion, or that of the metal coefficient was higher to avoid the tensile forces at the interface. If the difference in CTE was positive, i.e., metal alloy CTE _alloy_ > CTE _ceramic_, the component was in tension and the ceramic component was in compression. If it was negative, CTE _alloy_ < CTE _ceramic_, the forces were reversed (Table 3).

### 3.4. Hardness Tests

**Figure 6:**
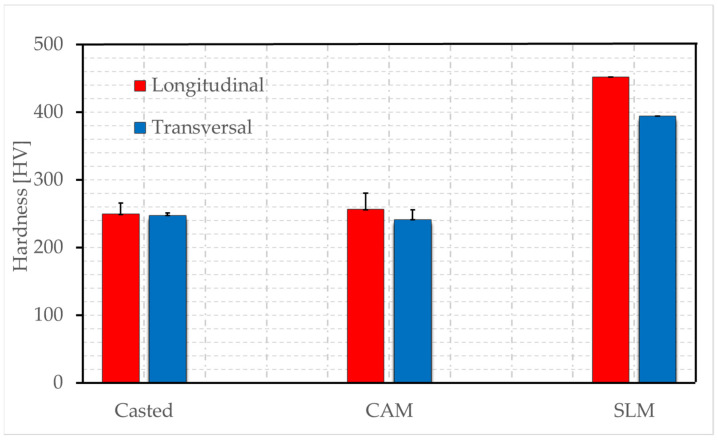
Comparative analysis of Vickers hardness tests.

The results obtained by Vickers hardness testing for each sample (3 samples for each type with 5 identifications) showed a very high hardness of the samples obtained by sintering (Figure 6, Table S1), compared with those obtained by the other two methods—casting and milling. Those two methods had relatively close Vickers hardness values for the two analyzed sections.

For the component obtained by selective laser melting, the hardness had nearly double values (453, 395 HV). Therefore, this manufacturing technology was the best from this point of view, providing durability, facilitating the formation of oxide film upon contact with the environment, and providing good adhesion with ceramics. 

### 3.5. Three-Poins Bending Test

**Figure 7:**
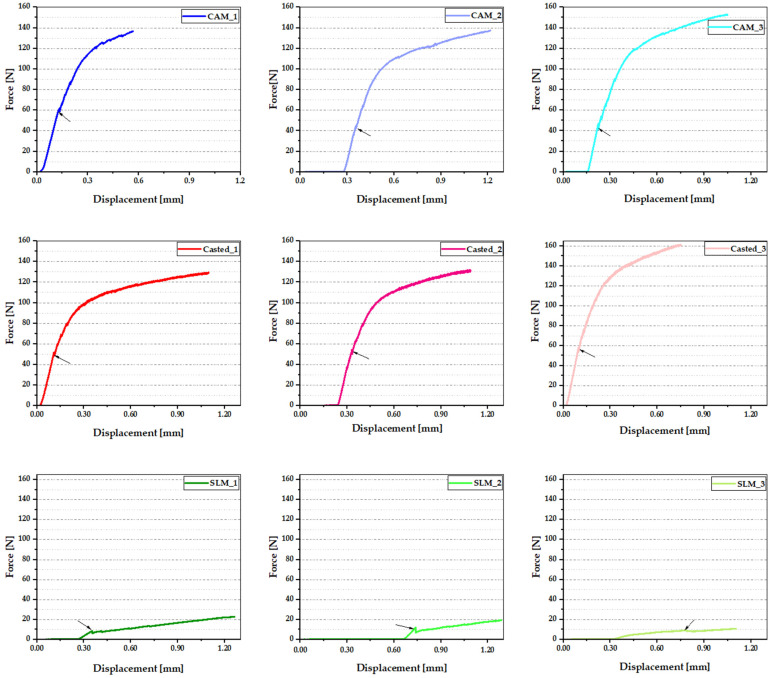
Crack initiation (marked with black arrow) on force-displacement graphs for each production technology.

**Figure 8:**
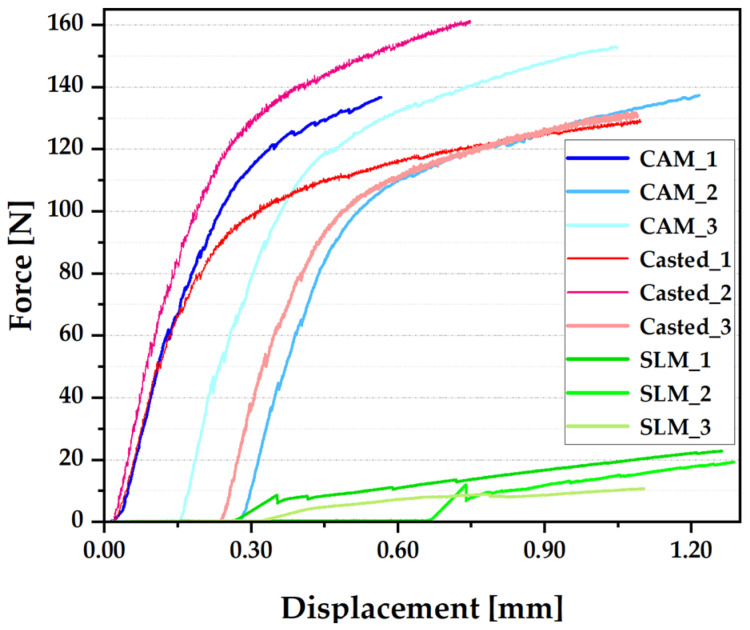
Comparative force-displacement graphs for the three methods of production.

Depending on the production technology, three data sets were applied, with curves that had quite small differences between them. The results of the three-point bending tests are shown as force-displacement curves, as shown in Figure 7, and compared as shown in Figure 8. The shades of red represent those corresponding to the parts obtained by the conventional method (casting); the shades of blue represent the curves related to the parts obtained by milling; and the shades of green represent the parts obtained by selective laser sintering. The first signs of cracking of the ceramic component, specific to each production technology, were marked with a black arrow.

For the castings, the force values were 51.14 N, 52.68 N, and 57 N, respectively, with an average force of approximately 53.6 N; for milled parts, the force values were 58.07 N, 41.84 N, and 42.29 N, respectively, with an average force of approximately 47.4 N; for the sintered parts, the force values were 80.43 N, 10.90 N, and 8.78 N, respectively, with an average force value of approximately 9.24 N (Figure 5).

The bending strength is the maximum point in the curve—the maximum value of the load borne by the material before breakage occurs. The average values were previously provided (Figure 8).

In general, metal–ceramic systems are designed to have superior bending strength to withstand mechanical stress, to ensure performance in the buccal environment, and to withstand mastication forces. Based on the analysis of these curves, it can be stated that casting is the manufacturing procedure that provides the best results, followed by CAM and by the SLM method.

## 4. Discussion

There is no doubt that, to guarantee aesthetic results, the strength of the metal–ceramic bond is a basic factor, as poor strength can lead to the early failure of a restoration, regardless of the success of the initial results. However, there is no agreement in the literature regarding the best adhesion mechanism between metal and ceramics [27,28,29,30]. There are many questions about test methods for evaluating metal–ceramic bonding, since the best method for accurately measuring this property is still unknown. Some authors state that there is no methodology capable of measuring shear forces along the metal–ceramic interface [28,31]. Regarding analyzed couples in which the greatest difference in CTE results have been reported, microscopic microcracks have been highlighted by microscopic analysis. The mechanical compatibility, chemical compatibility, and stress-distribution compatibility in metal–ceramic systems are critical factors for the successful integration of different materials in dental restorations [18]. This successful integration refers to the ability of the metal component and the ceramic to coexist without significant adverse interactions, such as chemical reactions, phase changes, or mechanical damage.

Regarding the micrographs that were obtained, the cast alloy had a heterogeneous microstructure—dendritic with defects and large pore volume—which can generate internal stresses, so that the bending strength of the cast Co–Cr alloy was low. The CAM sample showed superior microstructural homogeneity, and the sintered alloy illustrated the presence of dense structures with homogeneous granules, along with reduced porosity and defects. However, by analyzing the metal–ceramic interface, it was found that the uniform succession of layers, the almost perfect adhesion, and the fewest defects (both in the ceramic mass and the metal mass) were characteristics of the parts obtained by casting.

Vickers hardness tests indicated that the alloy obtained by the SLM method was preferred for metal–ceramic applications. High average values have a positive impact on the properties of the oxide film formed in the oral cavity, the durability of the system, and the adhesion of the ceramic.

The three-point bending test demonstrated that the elasticity moduli of Co–Cr alloys had reduced values. In general, metal–ceramic systems are designed to have superior bending strength to withstand mechanical stress and mastication forces.

Based on the analysis of these curves, it can be stated that casting is the manufacturing procedure that provides the best results, followed by milling (average value of approximately 148 kN) and by the SLM method (average value of approximately 22 kN).

The thermal cycle can create challenges in the compatibility of metal–ceramic systems, due to mismatches of the thermal expansion coefficients. Large differences in CTE can lead to the formation of internal stresses—even cracking—or lack of adhesion to the metal–ceramic interface. It is necessary that the metal and the porcelain have identical coefficients of thermal expansion, or that the thermal expansion coefficient of the metal be higher than that of the porcelain, to avoid tensile forces at the interface.

## 5. Conclusions

The manufacturing procedure for the metal component of a metal–ceramic system can have a significant impact on the strength of the bond of the system. Based on the studies that were carried out, the most appropriate manufacturing procedure for the Co–Cr alloy, to ensure the compatibility of metal–ceramic systems for dental restorations, was casting, although each of the methods studied had its advantages and disadvantages. The choice of method should be based on the specific requirements of a particular restoration, as well as on the properties of the materials used. It is possible that even in the case of metal–ceramic couples where no cracks are revealed, if the sample is subjected to a wear test, cracks may appear, due to intrinsic stresses at the metal–ceramic interface. (This situation is a simulation of the mastication process.) Given this assumption, it is probable that dynamic evaluations are indicated for the actual evaluation of metal–ceramic bonding. There is a need to develop a standard for the dynamic testing of metal–ceramic couples.

## Figures and Tables

**Table 1:** Chemical composition of the Co–Cr alloy used in the experimental procedure.

Alloy	Element						
	Co	Cr	Mo	W	Mn	Si	N
**Herenium**	59.0	25.0	4.0	10.0	0.8	1.0	0.2

**Table 2:** Thermal expansion coefficients of the samples’ function of production technology.

Temperature Range [°C]	Coefficient of Thermal Expansion (×10^−6^/°C)		
	CAM	SLM	CASTING
**20–300**	13.31	12.79	14.02
**300–600**	18.23	13.93	12.25
**600–900**	19.41	18.91	15.75
**900–1200**	23.38	20.39	17.15

**Table 3:** Thermal expansion coefficients of the ceramic parts.

**Temperature Range [** **°C]**	**Coefficient of Thermal Expansion (×10^−6^/** **°** **C)**	
	**Enamel**	**Dentine**
**20–300**	15.1	13.5
**300–600**	17.4	14.8
**600–900**	22.1	20.4

## Supplementary Materials

The following supporting information can be downloaded at: https://www.mdpi.com/article/10.3390/ma16165556/s1, Table S1. Vickers hardness test results.
