# Supplementary material for: Metal–Ceramic Compatibility in Dental Restorations According to the Metallic Component Manufacturing Procedure
Source: Materials (Basel). 2023 Aug 10;16(16):5556. doi: 10.3390/ma16165556 (PMC10456282; doi:10.3390/ma16165556)
Supplement: Supplementary file 1 [file materials-16-05556-s001.zip › materials-2536749-supplementary.pdf]

Table S1. Vickers hardness test results

Sample/method of obtaining	Hardness [HV5]	Mean value [HV5]	Standard deviation	Section
Cast Co-Cr	267	250	16	Longitudinal
	246			
	237			
CAM Co-Cr	258	257	3	
	259			
	254			
SLM Co-Cr	459	453	14	
	452			
	449			
Cast Co-Cr	253	248	13	Transversal
	257			
	233			
CAM Co-Cr	245	242	5	
	245			
	236			
SLM Co-Cr	371	395	23	
	395			
	417			
